# Tuberculosis sequelae: a rare clinical medicine

**DOI:** 10.11604/pamj.2025.52.100.48609

**Published:** 2025-11-10

**Authors:** Ashwin Karnan

**Affiliations:** 1 Department of Respiratory Medicine, Datta Meghe Institute of Higher Education and Research, Sawangi (Meghe), Wardha, Maharashtra, India

**Keywords:** Tuberculosis, pleural effusion, fibrothorax, pleural calcification

## Image in medicine

A 59-year-old male, a farmer and a chronic alcoholic, presented to the emergency department with complaints of breathing difficulty, cough with expectoration, and fever for the past 3 days. The patient gives a history of tubercular pleural effusion 3 years back, for which he took treatment for 9 months. Laboratory investigations were within normal range. Chest X-ray showed patchy opacities in the bilateral upper lobes and localised left lower pleural calcification. High-resolution computed tomography (HRCT) of the patient’s thorax showed centrimetric mediastinal lymph nodes, bilateral upper lobe consolidation with cystic bronchiectatic changes, and calcified pleural plaques on the left side, with small hydropneumothorax, with a communicating peripheral bronchus suggestive of bronchopleural fistula. After ruling out active tuberculosis by sputum analysis and bronchoscopy, the patient was referred to the pulmonology department, where he underwent bronchoscopic coil application for bronchopleural fistula closure. The patient was treated with broad-spectrum antibiotics, nutritional support, and physiotherapy. The patient improved symptomatically and is currently on follow-up. Despite efficient treatment modalities, some patients develop consequences. The most common parenchymal sequelae are cavitation, fibrotic bands, aspergilloma, cicatrisation, and end-stage lung disease. The pleural sequelae include chronic empyema, fibrothorax, bronchopleural fistula, and pneumothorax. The airway sequelae include bronchiectasis, tracheobronchial stenosis, and broncholithiasis. The vascular sequelae include pulmonary arteritis, thrombosis, bronchial arteritis, and Rasmussen aneurysm. Early diagnosis and treatment, ensuring treatment adherence, monitoring response to treatment, pulmonary rehabilitation, and regular follow-ups have to be ensured to prevent consequences.

**Figure 1:**
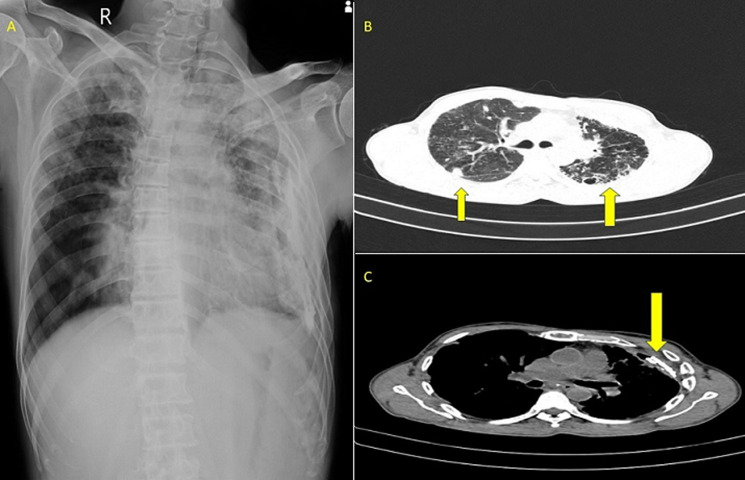
A) bilateral upper lobe opacities with left pleural thickening; B) yellow arrow showing bilateral upper lobe consolidation with cystic bronchiectatic changes; C) yellow arrow showing left calcified pleural plaques

